# *Mycoplasma hyorhinis*-Contaminated Cell Lines Activate Primary Innate Immune Cells via a Protease-Sensitive Factor

**DOI:** 10.1371/journal.pone.0142523

**Published:** 2015-11-13

**Authors:** Simon Heidegger, Alexander Jarosch, Martina Schmickl, Stefan Endres, Carole Bourquin, Christian Hotz

**Affiliations:** 1 Center for Integrated Protein Science Munich (CIPSM), Division of Clinical Pharmacology, Medizinische Klinik und Poliklinik IV, Ludwig-Maximilians-Universität München, 80336 Munich, Germany; 2 III. Medizinische Klinik, Klinikum Rechts der Isar, Technische Universität München, 81675 Munich, Germany; 3 Chair of Pharmacology, Department of Medicine, Faculty of Science, University of Fribourg, 1700 Fribourg, Switzerland; Universitatsklinikum Freiburg, GERMANY

## Abstract

*Mycoplasma* are a frequent and occult contaminant of cell cultures, whereby these prokaryotic organisms can modify many aspects of cell physiology, rendering experiments that are conducted with such contaminated cells problematic. Chronic *Mycoplasma* contamination in human monocytic cells lines has been associated with suppressed Toll-like receptor (TLR) function. In contrast, we show here that components derived from a *Mycoplasma hyorhinis*-infected cell line can activate innate immunity in non-infected primary immune cells. Release of pro-inflammatory cytokines such as IL-6 by dendritic cells in response to *Mycoplasma hyorhinis*-infected cell components was critically dependent on the adapter protein MyD88 but only partially on TLR2. Unlike canonical TLR2 signaling that is triggered in response to the detection of *Mycoplasma* infection, innate immune activation by components of *Mycoplasma*-infected cells was inhibited by chloroquine treatment and sensitive to protease treatment. We further show that in plasmacytoid dendritic cells, soluble factors from *Mycoplasma hyorhinis*-infected cells induce the production of large amounts of IFN-α. We conclude that *Mycoplasma hyorhinis*-infected cell lines release protein factors that can potently activate co-cultured innate immune cells via a previously unrecognized mechanism, thus limiting the validity of such co-culture experiments.

## Introduction

*Mycoplasma* are a heterogeneous group of wall-less, self-replicating prokaryotes with great diversity in replication rates and culture requirements. Some species, such as *Mycoplasma hyorhinis*, are found as a frequent and occult contaminant of both human and murine cell cultures. These organisms can modify many aspects of cell physiology, rendering the interpretation of experiments that are conducted with contaminated cells problematic [1]. Because of their slow growth and resistance against many antibiotics, *Mycoplasma* contamination of cell cultures can progress undiscovered over a long period of time and thus represents a significant problem in experimental research [2].

For the detection of pathogens, cells of the innate immune system are equipped with specialized pattern-recognition receptors (PRRs). These are germline-encoded receptors, which detect conserved molecular structures that are specific to invading microbes but are not present in uninfected host cells. Recognition of such pathogen-associated molecular patterns (PAMPs) leads to activation and maturation of antigen-presenting cells, release of pro-inflammatory cytokines and the initiation of a subsequent adaptive immune response. Toll-like receptors (TLRs) constitute a family of trans-membrane PRRs that are broadly expressed in non-hematopoietic and hematopoietic cells such as dendritic cells (DCs) [3]. TLR ligation by a variety of microbial components including lipopolysaccharides (LPS, TLR4) or DNA-containing CpG motifs (TLR9) leads to activation of antigen-presenting cells, production of pro-inflammatory cytokines and the release of type I interferon (IFN-α and IFN-β) [4]. Downstream signaling of TLRs is mediated by the adaptor proteins MyD88 (all TLRs except TLR3) and TRIF (TLR3 and 4) [5]. Some TLRs such as TLR2 are localized on the cell surface and predominately recognize microbial membrane components, whereas other TLRs such as TLR9 are expressed within endosomes and mainly detect nucleic acids [3].

*Mycoplasma*-derived lipoproteins can be detected by both the human and murine innate immune system through ligation to the outer plasma membrane-bound TLR2 and subsequent downstream signaling via MyD88 [6]. Studies in primary murine macrophages [7] as well as human embryonic kidney cell lines [8] have shown that heterodimerization with either TLR1 or TLR6 can augment TLR2 activity and allows for the discrimination of different lipoproteins. Ligation of *Mycoplasma*-derived lipoproteins by TLR2 on murine DCs can result in potent cytokine release and activation of bystander cells [9]. In mice, bone marrow-derived DCs that genetically lack functional TLR2 showed abolished IL-6 production in response to *Mycoplasma pulmonis* [10], which translated into reduced resistance of TLR2-deficient mice against pulmonary infection with live *M*. *pulmonis*.

A previous report has shown that chronic *Mycoplasma* contamination of the human monocytic cell line THP-1 can suppress its responsiveness to various TLR stimuli [11]. Such immortalized cells and other tumor cell lines are commonly used to investigate their interaction with immune cells. Primary immune cells of both murine and human origin are often used in co-culture experiments together with cell lines and could thus be affected by *Mycoplasma* without being directly infected. Indeed, immune cells are poised to sense infection in surrounding cells and to react rapidly to such a threat. In this study, we investigated whether *Mycoplasma*-infected cell lines can affect primary bystander cells. We demonstrate that one or more proteinase-sensitive components derived from *Mycoplasma hyorhinis*-infected cell lines can activate bystander, non-infected innate immune cells to produce pro-inflammatory cytokines independently of canonical TLR2 signaling as well as type-I interferon by pDC, which both can strongly corrupt results obtained by co-culturing experiments.

## Materials and Methods

### Ethics statement

This study was carried out in strict accordance to the guidelines of the German animal protection law (TierSchG). All procedures including housing and sacrifice were performed as authorized by and were reported to the responsible state office Regierung von Oberbayern (ROB, Germany) per standard legal procedure. Under this law, no special permit was necessary for isolating organs from mice after they have been sacrificed by cervical dislocation following isoflurane anesthesia. No manipulations were performed and no samples were collected prior to euthanasia.

### Mice

Female C57BL/6 mice were purchased from Harlan-Winkelmann and Janvier. TLR2-deficient mice (TLR2^-/-^) were a generous gift from Dirk H. Busch (Munich) and have been described previously [12]. All mice were at least 8 weeks of age at the time of bone marrow harvest.

### Cell lines

The murine B16-F10 (B16) melanoma cell line, CT26 colon carcinoma cell line and EL4 lymphoma cell line are commercially available (ATCC, Wesel, Germany) and have been described previously [13–15]. Tumor cell lines were cultured in DMEM supplemented with 10% FCS, 2 mM L-glutamine and 100 μg/ml streptomycin and 1 IU/ml penicillin. The contaminating *Mycoplasma* species was identified by commercial PCR multiplex testing (Multiplexion, Heidelberg, Germany) as described previously [16]. For the eradication of *Mycoplasma*, B16 cells were cultured in the presence of 25 μg/ml Plasmocin (Invivogen, Toulouse, France) for 14 days. Cells were split every 2–3 days. Culture supernatants from passages 5–15 were centrifuged (400 G, 8 min) and were subsequently used for stimulation experiments. The culture supernatant was mixed with fresh complete RPMI medium (10% FCS, 2 mM L-glutamine, 100 μg/ml streptomycin and 1 IU/ml penicillin) at a 1:1 ratio if not stated otherwise. Fresh complete DMEM medium mixed with RPMI at a similar ratio was used as negative control. In some experiments the culture supernatant was filtered through a 0.22 μm filter (Merck Millipore, Billerica, MA) and/or treated with UV irradiation (254 nm, 40.000 μJ/cm^2^; CL-1000 cross-linker from UVP, Upland, CA). To obtain lysates, cells were snap-frozen in liquid nitrogen at a density of approximately 1-2x10^7^ cells/ml and thawed on ice prior to stimulation.

### Generation of bone marrow-derived dendritic cells

Bone marrow cells were harvested from murine femur and tibia and erythrocytes were lysed with ammonium chloride buffer (BD Biosciences, Heidelberg, Germany). For bone marrow-derived GM-CSF DCs, mouse bone marrow cells were cultured in complete RPMI 1640 (10% FCS, 2 mM L-glutamine, 100 μg/ml streptomycin and 1 IU/ml penicillin) supplemented with 20 ng/ml GM-CSF and 20 ng/ml IL-4 (Tebu Bio, Offenbach, Germany). On day 6 to 7, cells were harvested. Plasmacytoid DC (pDC)-containing Flt3-DC were generated from bone marrow cells cultured in complete RPMI 1640 supplemented with 1 mM sodium pyruvate, 1% non-essential amino acids (MEM-NEAA), 3.75 x 10^−4^% 2-mercaptoethanol and 20 ng/ml recombinant Flt3-L (Peprotech, London, UK or Tebu Bio, Offenbach, Germany). On day 7 to 9, cells were harvested and B220^+^ pDCs were isolated with magnetic microbeads (Miltenyi Biotec, Bergisch-Gladbach, Germany or BD iMAG, BD Biosciences, Heidelberg, Germany) according to the manufacturer’s protocol.

### Cell culture, stimulation and inhibitors

Murine immune cells were cultured in complete RPMI. LPS (5 μg/ml), FSL-1 (500 ng/ml) was purchased from Invivogen. MALP-2 (500 ng/ml) was from Novus Biologicals (Cambridge, UK). The PTO-modified CpG oligonucleotide 1826 (CpG, 3 μg/ml, 5’-TCCATGACGTTCCTGACGTT-3’) was synthesized by Eurofins MWG Operon (Ebersberg, Germany). The inhibitor of endosomal TLR activity chloroquine (from Invivogen) was used at a concentration of 5 μM, if not indicated otherwise. For stimulation of primary cells, supernatant from infected cells was mixed at a ratio of 1:1 with fresh medium. Cell lysates were mixed to responder cells at a ratio of 1:100 if not stated otherwise. For protein digestion, cell lysates were treated with 16 U/ml proteinase K (Sigma-Aldrich, Munich, Germany) at 55°C for 1 h with constant shaking. Enzymatic digestion of nucleid acids with RNase A and DNase I (both from Sigma-Aldrich) was performed according to the manufacturer’s protocol.

### Quantification of cytokines

The concentration of IL-1β, IL-6, IL-10 and IL-12p70 in the culture supernatant was determined by enzyme-linked immunosorbent assay (ELISA) according to the manufacturers’ instructions (BD Biosciences and BioLegend, San Diego, CA, USA). IFN-α was measured as described previously [17]. In brief, rat monoclonal antibody to mouse IFN-α (clone RMMA-1) was used as the capture antibody, rabbit polyclonal antibody to mouse IFN-α for detection (both from PBL Biomedical Laboratories, Piscataway, NJ) together with HRP-conjugated donkey antibody to rabbit IgG as the secondary reagent (Jackson ImmunoLaboratories, Bar Harbor, ME). Recombinant mouse IFN-α (PBL Biomedical Laboratories) was used as standard (IFN-α concentration in IU/ml).

### Quantitative real-time PCR

Total RNA was isolated from bone marrow cells lysed with TRIzol reagent (Ambion, Waltham, MA) and was subsequently transcribed with the SuperScript III Reverse Transcriptase (Thermo Fischer Scientific, Waltham, MA) according to the manufacturers’ protocol. The specific primer pairs were as follows: mIL-6 fwd GTAGCTATGGTACTCCAGAAGAC, rev ACGATGATGCACTTGCAGAA; mActin fwd CACACCCGCCACCAGTTCG, rev CACCATCACACCCTGGTGC. The qPCR Core kit for SYBR Green I (Eurogentec, Köln, Germany) and a LightCycler 480 II (Roche, Mannheim, Germany) real-Time PCR system were used for the analysis.

### Statistics

All data are presented as mean + S.E.M. Statistical significance of single experimental findings was assessed with the independent two-tailed Student’s t-test. For multiple statistical comparison of a data set, the one-way ANOVA test with Bonferroni post-test was used. Significance was set at *p*-values *p* < 0.05, *p* < 0.01 and *p* < 0.001 and was then indicated with an asterisk (*, ** and ***). All statistical calculations were performed using Graphpad Prism (GraphPad Software, San Diego, USA).

## Results

### *Mycoplasma*-infected B16 cells stimulate production of pro-inflammatory cytokines in primary immune cells

**Fig 1:**
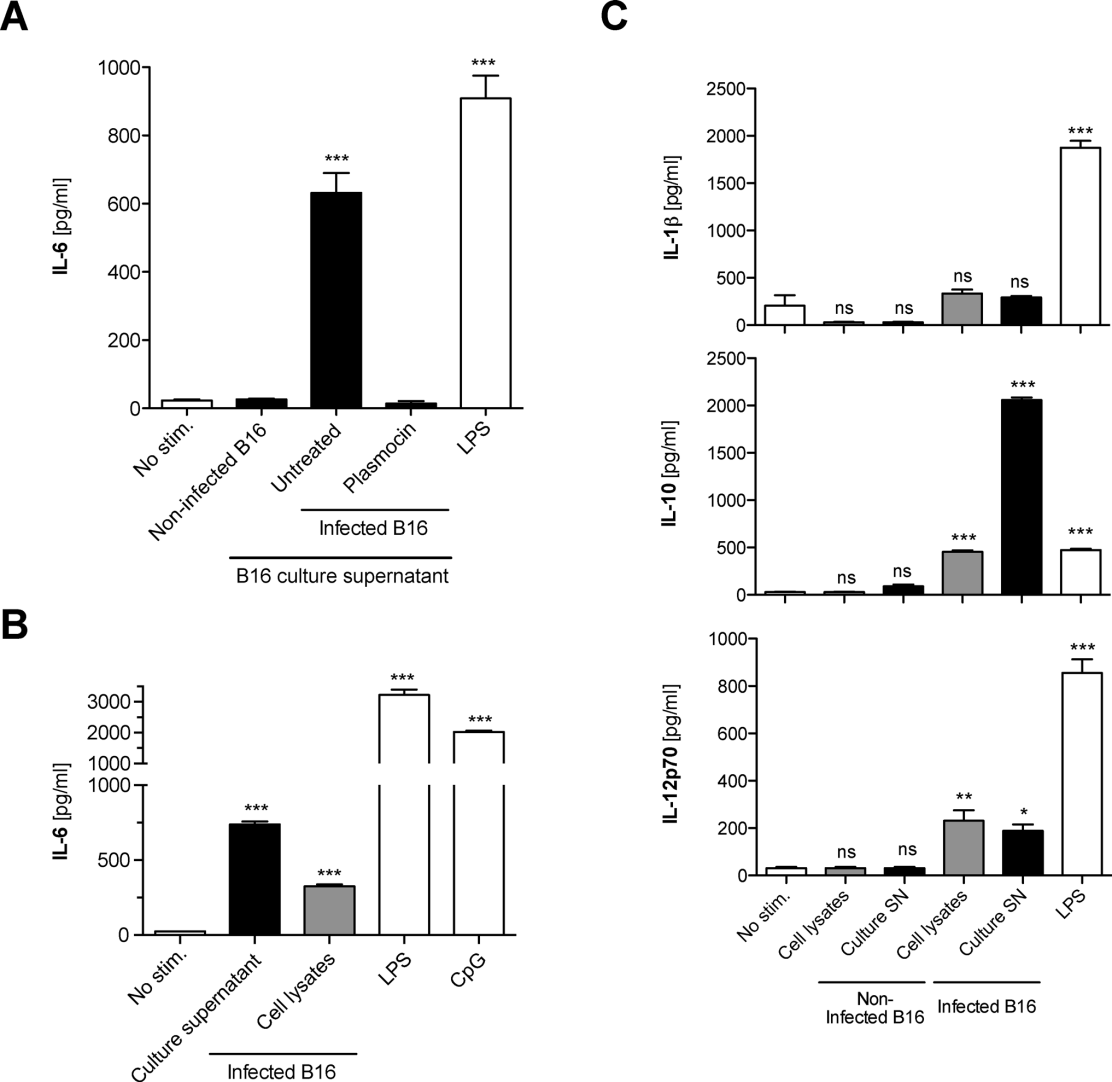
*Mycoplasma hyorhinis*-infected B16 melanoma cells stimulate production of proinflammatory cytokines by primary immune cells. Freshly isolated murine bone marrow cells were cultured in the presence of **(A)** the diluted cell culture supernatant (black bars) or **(B)** cell lysates (grey bars) of either a *Mycoplasma hyorhinis*-infected or non-infected B16 melanoma cell line. After 18 h, levels of IL-6 in the bone marrow cell culture supernatant were analyzed by ELISA. The TLR4 ligand LPS and the TLR9 ligand CpG were used as positive controls. In one condition, supernatant from formerly infected B16 cells in which *Mycoplasma hyorhinis* had been eradicated with the antibiotic Plasmocin, was used. **(C)** Bone marrow cells were cultured as in (B) in the presence of either culture supernatant or cell lysates of *Mycoplasma hyorhinis*-infected B16 cells. The levels of IL-10, IL-12p70 and IL-1β were analyzed in the bone marrow cell culture supernatant by ELISA after 18 h. All data give the mean + S.E.M. of triplicate samples and are representative of at least three independent experiments. Asterisks indicate statistically significant differences to the non-stimulated control. ns, not significant; SN, supernatant.

To investigate whether *Mycoplasma*-infected cell lines can affect bystander cells, freshly isolated murine bone marrow cells, which harbor an abundance of different precursor and mature immune cells, were cultured in the presence of supernatants or cell lysates from *Mycoplasma hyorhinis*-infected B16-F10 murine melanoma cells (B16). Bone marrow cells that were supplemented with the supernatant from contaminated B16 cell cultures produced large amounts of the pro-inflammatory cytokine IL-6 **(Fig 1A)**. This effect was not restricted to the B16 melanoma line, since culture supernatant or cell lysates from *Mycoplasma*-contaminated CT26 colon carcinoma cells or the EL4 lymphoma cell line also resulted in potent IL-6 release from co-cultured bone marrow cells **(S1 Fig)**. Culture supernatant from B16 cells in which *Mycoplasma* had been eradicated by treatment with the antibiotic Plasmocin induced only trace cytokine release, which was comparable to the background level induced by supernatant from uninfected B16 cells. Activation of bone marrow cells was induced not only by culture supernatant but also by cell lysates of infected B16 cells **(Fig 1B)**. The well-characterized TLR ligands LPS and CpG were used as positive controls. *Mycoplasma hyorhinis*-infected B16 cells also induced production of IL-10 and IL-12p70 but not IL-1β in co-cultured bone marrow cells **(Fig 1C)**. The infected B16 cells did not release any of these cytokines themselves (data not shown). In conclusion, *Mycoplasma hyorhinis*-infected cell lines harbor and release a factor that can stimulate bystander immune cells to produce pro-inflammatory cytokines.

### Innate immune activation by a *Mycoplasma*-infected cell line is dependent on MyD88 signaling

**Fig 2:**
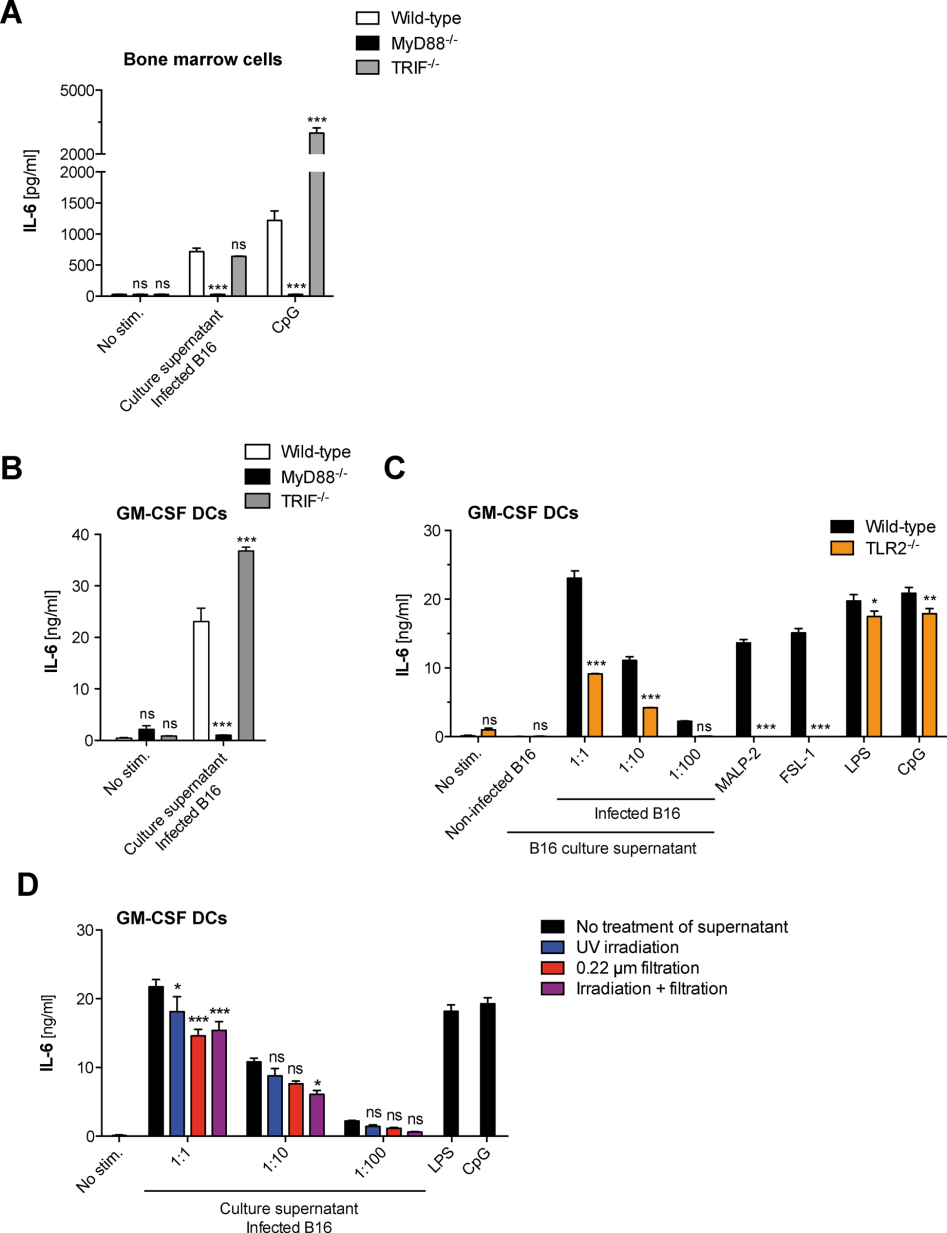
Innate immune activation by *Mycoplasma hyorhinis*-infected B16 cells is fully dependent on MyD88 signaling but only partially on TLR2. **(A)** Bone marrow cells or **(B)** differentiated GM-CSF DCs from wild-type, MyD88- or TRIF-deficient mice were cultured in the presence of diluted supernatant from *Mycoplasma hyorhinis*-infected B16 cells for 18 h. IL-6 levels in the immune cell culture supernatant were analyzed by ELISA. **(C)** GM-CSF DCs from wild-type and TLR2-deficient mice were cultured as in (A) in the presence of diluted supernatant from *Mycoplasma hyorhinis*-infected B16 cells and IL-6 levels in the DC culture supernatant were analyzed by ELISA. **(D)** GM-CSF DCs from wild-type mice were cultured as in (A). In some conditions, the diluted B16 culture supernatant was decontaminated by filtration (red bars), UV irradiation (blue bars) or both (purple bars) before it was added to the DC culture. IL-6 levels in the DC culture supernatant were analyzed by ELISA after 18 h. Data give the mean + S.E.M. of triplicate samples and are representative of at least two independent experiments. Asterisks indicate statistically significant differences to the appropriate wild-type control (in A-C) or to the appropriate non-decontaminated conditions (in D).

As the detection of common pathogen-associated molecular patterns including *Mycoplasma*-derived lipoproteins is largely dependent on TLRs and subsequent downstream signaling via MyD88 and TRIF [3], we examined whether these two adapter proteins were critical for the detection of the active components released from a *Mycoplasma hyorhinis*-infected cell line by innate immune cells in our setting. In addition to primary bone marrow cells, we analyzed cytokine production by dendritic cells (DCs), which form a specialized subset of antigen-presenting cells and are potent producers of pro-inflammatory cytokines in response to various microbial stimuli. Both wild-type bone marrow cells and differentiated DCs (GM-CSF DCs) produced IL-6 in response to culture supernatant of *Mycoplasma hyorhinis*-infected B16 cells **(Fig 2A and 2B).** The release of IL-6 was greatly reduced in bone marrow cells and DCs from MyD88-deficient mice. In contrast, deficiency for the TLR3/4 adapter protein TRIF did not inhibit but rather enhanced cytokine secretion in response to *Mycoplasma hyorhinis*-infected cell culture material. Indeed, TRIF has been described as a negative regulator of MyD88-dependent TLR signaling, as cytokine release downstream of MyD88 is strongly increased in TRIF-deficient mice [18, 19].

As MyD88 is a central adapter protein to TLRs with both endosomal and cell surface localization, we next investigated in which cellular compartment *Mycoplasma*-infected cell components are recognized. Bone marrow cells were treated with chloroquine, which is commonly used as an inhibitor of endosomal TLRs [20, 21]. Treatment with chloroquine largely inhibited the release of IL-6 by bone marrow cells in response to *Mycoplasma hyorhinis*-infected cell line components or the TLR9 ligand CpG **(S2A Fig)**In contrast, chloroquine did not block IL-6 production by bone marrow cells or GM-CSF DCs following stimulation with either the synthetic lipoprotein TLR2 ligand FSL-1 or LPS **(S2B and S2C Fig)**, which both activate surface-localized TLRs.

MyD88 does not only mediate the downstream signaling of most TLRs but also of the IL-1 receptor family, whose members are commonly activated by caspase-1-dependent cleavage [3]. Treatment of bone marrow cells with the pan-caspase inhibitor zVAD did not influence IL-6 release in response to culture supernatant of *Mycoplasma hyorhinis*-infected B16 cells (data not shown) indicating that immune cell activation was independent of bio-active IL-1 family members. Taken together, these data show that sensing of components from *Mycoplasma hyorhinis*-contaminated cell lines is mediated by MyD88 and is sensitive to the endosomal TLR inhibitor chloroquine.

### Immunostimulation by *Mycoplasma hyorhinis*-infected B16 cells occurs in the absence of active infection and is only partially dependent on TLR2 signaling

As the detection of known *Mycoplasma*-derived lipoproteins is largely dependent on heterodimerization of TLR2 with either TLR1 or TLR6 and subsequent downstream signaling via MyD88 [6, 7, 22], we examined whether TLR2 was critical for the detection of the active components released from a *Mycoplasma*-infected cell line in our setting. In GM-CSF DCs that genetically lack TLR2, the induction of IL-6 in response to culture supernatant from *Mycoplasma hyorhinis*-infected B16 cells was only reduced by 50% **(Fig 2C)**. In sharp contrast, IL-6 stimulation with MALP-2 and other defined TLR2 agonists was completely abrogated in absence of functional TLR2 signaling. These data demonstrate that the culture supernatant from *Mycoplasma hyorhinis-*infected B16 cells contains both TLR2-dependent and TLR2-independent immunostimulatory factors that activate bystander immune cells via the MyD88 pathway.

We next determined whether the immunostimulatory capacity of culture supernatant from *Mycoplasma hyorhinis*-infected B 16 cells required active infection of target immune cells. Generally, when non-infected B16 cells were transiently cultured in the presence of supernatant from *Mycoplasma hyorhinis*-contaminated cell lines, they were subsequently infected and acquired the ability to induce production of IL-6 in primary immune cells **(S3 Fig)**. To examine whether infection of the DC culture by the contaminating *Mycoplasma* species was necessary for immune activation, the supernatant from *Mycoplasma hyorhinis-*infected B16 cells was treated by UV irradiation and/or filtration. Such decontaminated B16 culture supernatants were no longer infectious **(S3 Fig),** but still induced strong IL-6 release in GM-CSF DCs **(Fig 2D)**. These data show that the immune response in DCs to culture supernatant from *Mycoplasma hyorhinis*-contaminated cell lines is not dependent on active infection.

### Innate immune activation by *Mycoplasma hyorhinis*-infected B16 cells is mediated by a protein factor

**Fig 3:**
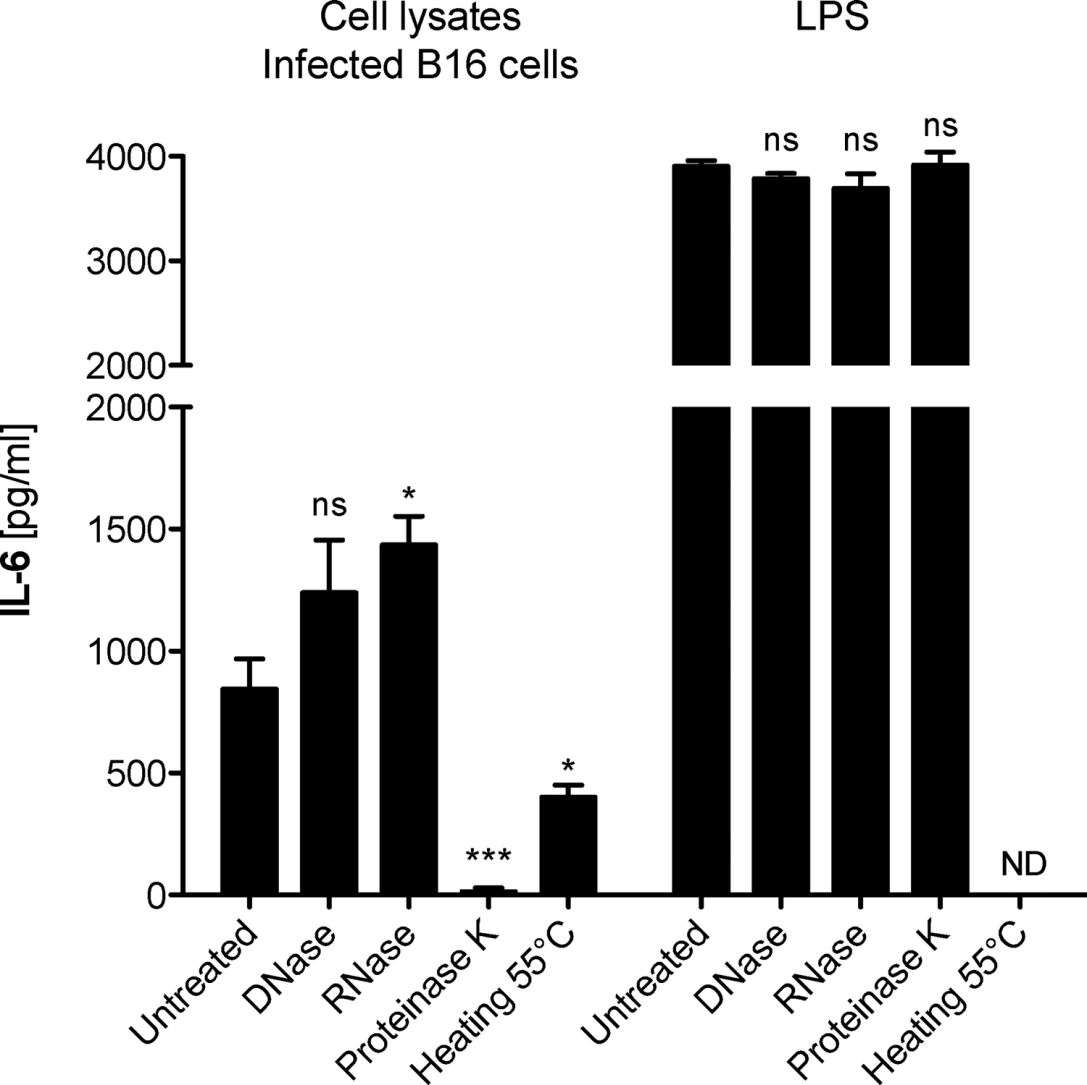
Innate immune activation by *Mycoplasma hyorhinis*-infected B16 cells is mediated by a protein factor. Bone marrow cells were cultured in the presence of cell lysates of *Mycoplasma hyorhinis*-infected B16 cells. Some cell lysates were pre-treated either with DNase, RNase or proteinase K. 18 h later, IL-6 levels in the bone marrow cell culture supernatant were analyzed by ELISA. Protein digestion was performed at 55°C. In one condition, B16 cell lysates were heated to 55°C in the absence of proteinase K. Data give the mean values of triplicate samples + S.E.M. and are representative of two independent experiments. Asterisks indicate statistically significant difference to the appropriate non-inhibitor-treated conditions. ND, not determined.

Endosomal TLRs are considered to detect mainly bacterial and viral nucleic acids. The ligation of *Mycoplasma*-derived lipoproteins to TLR2 and its heterodimers with either TLR1 or TLR6 has been shown to be critically dependent on the ligand’s lipid moiety since digestion with lipases but not proteases impaired the lipoproteins’ immunostimulatory capacity in human monocytic cells [23]. To determine the nature of the component responsible for immune cell activation by *Mycoplasma hyorhinis*-infected cells, B16 cell lysates were treated either with DNase I, RNase A or proteinase K before they were added to a bone marrow cell culture. Digestion of nucleic acids within the B16 lysates did not decrease the capacity of infected cells to induce IL-6 release from immune cells **(Fig 3)**. In contrast, protein digestion with proteinase K at 55°C resulted in strong inhibition of immunostimulation by *Mycoplasma*-contaminated cell lysates. Proteinase-treated B16 lysates failed to induce IL-6 production in bone marrow cells as determined by ELISA from the culture supernatant (**Fig 3**). To rule out that a spillover of enzyme to the bone marrow cell culture was responsible for the decrease in IL-6 protein, LPS-stimulated bone marrow cells were additionally supplemented with proteinase K-treated mock supernatants, which did not result in a decrease of IL-6 secretion. To further substantiate that the ligand itself is degraded by protease treatment, IL-6 mRNA expression was determined by quantitative real-time PCR. We found that proteinase K treatment of contaminated B16 cell lysates resulted in strongly impaired upregulation of IL-6 mRNA levels in bone marrow cells **(S4 Fig)** in contrast to LPS (not shown). In the absence of proteinase K, heating to 55°C resulted in less pronounced reduction in immunostimulatory capacity of *Mycoplasma*-infected cells. In conclusion, these data demonstrate that *Mycoplasma hyorhinis*-infected cell lines harbor a factor for which the pro-inflammatory activity is dependent on a protein component.

### Plasmacytoid dendritic cells produce IFN-α and IL-6 in response to lysates of *Mycoplasma hyorhinis*-infected B16 cells

**Fig 4:**
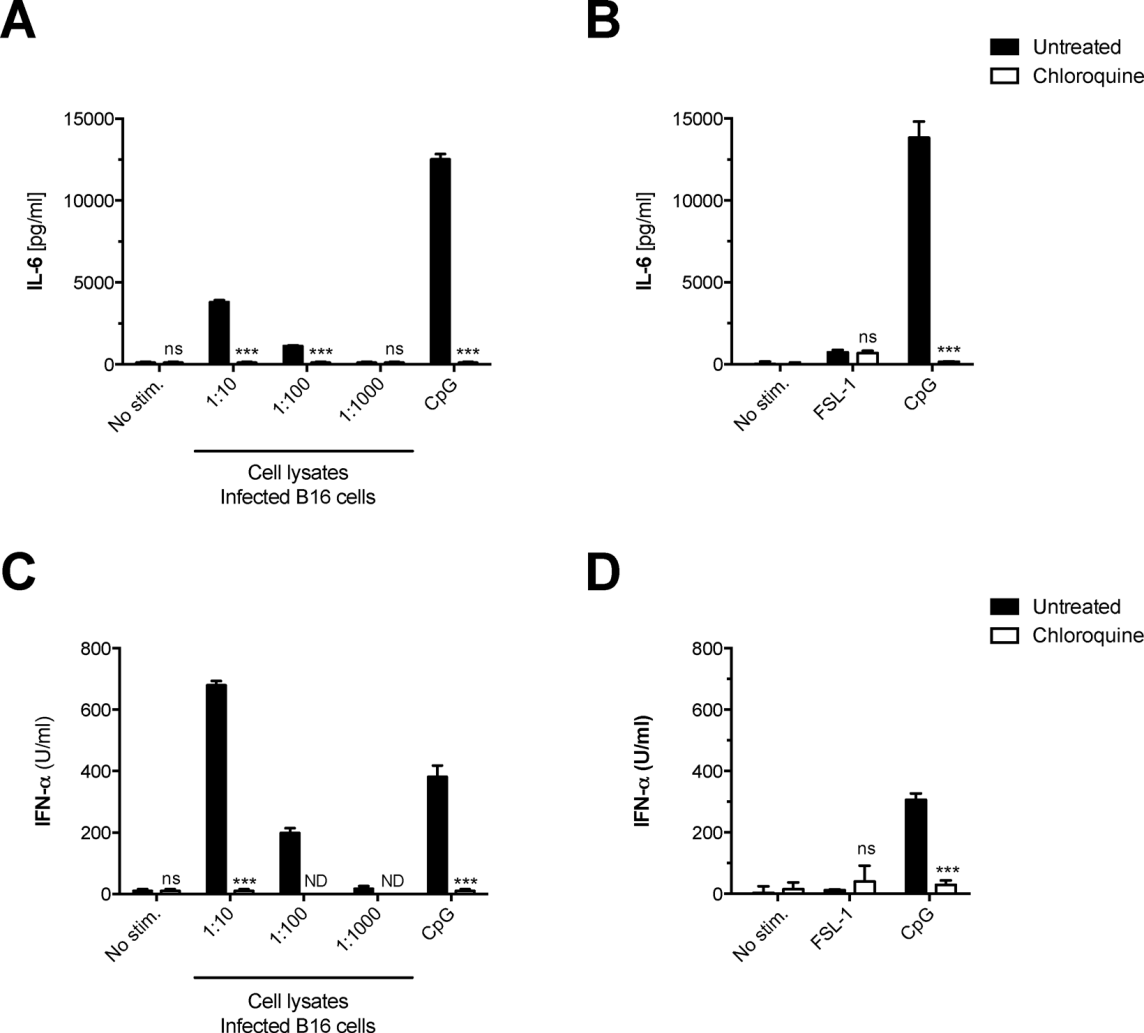
Plasmacytoid dendritic cells produce IFN-α and IL-6 in response to *Mycoplasma hyorhinis*-infected B16 cells. Bone marrow-derived plasmacytoid DCs were cultured in the presence of decreasing concentrations of lysates derived from *Mycoplasma hyorhinis*-infected B16 cells **(A, C)** or different TLR stimuli **(B, D)**. In some conditions, chloroquine was added to the pDC cultures. After 18 h, IL-6 and IFN-α levels in the pDC culture supernatant were analyzed by ELISA. Data give the mean + S.E.M. of triplicate samples and are representative of at least two independent experiments. Asterisks indicate statistically significant differences to the appropriate wild-type or untreated conditions.

Dendritic cells can be divided into several subpopulations. In contrast to conventional DCs (cDCs), that represent the main population in GM-CSF DC cultures, plasmacytoid DCs (pDC) morphologically resemble plasma cells and their function strongly depends on endosomal pathogen recognition via TLR7 and TLR9 [24]. To elucidate the role of pDCs in innate immune activation by components of *Mycoplasma*-infected cell lines, we analyzed the triggered cytokine release from bone marrow-derived pDCs. Following incubation with cell lysates from *Mycoplasma hyorhinis*-infected B16 cells, pDCs showed production of IL-6 that was entirely blocked by chloroquine treatment **(Fig 4A)**. In contrast, pDCs did not respond to stimulation with the synthetic lipoprotein TLR2 ligand FSL-1 **(Fig 4B)**. These data suggest that the immunostimulatory component from *Mycoplasma hyorhinis*-infected cells is recognized by pDCs within their endosomal compartment and is, as with cDC, distinct from canonical TLR2-dependent *Mycoplasma*-associated pathogenic patterns.

Functionally, pDCs are characterized by their ability to produce large amounts of type I interferons (IFNs) upon different stimuli such as viral or bacterial nucleic acids [24]. Culture supernatant from *Mycoplasma hyorhinis*-infected B16 cells induced concentration-dependent production of IFN-α in pDCs that was abolished following chloroquine treatment (**Fig 4C**). The synthetic lipoprotein TLR2 ligand FSL-1 did not induce IFN-α release by pDCs (**Fig 4D**). In summary, pDCs release both pro-inflammatory cytokines and type-I IFN in response to *Mycoplasma hyorhinis*-contaminated cell lines but not to canonical TLR2 PAMPs.

## Discussion

We show here that *Mycoplasma hyorhinis* contamination in tumor cell lines can substantially activate co-cultured bystander immune cells through a factor activating the MyD88 pathway. Further, we have discovered a previously unknown crosstalk of *Mycoplasma hyorhinis*-infected cells with pDCs leading to the secretion of IFN-α. Many insightful *in vitro* studies have been conducted to analyze the interaction of tumor cells with their immune cell microenvironment [25, 26]. Because of the important regulatory potential of type I interferons and other induced cytokines, we demonstrate that it is crucial to exclude *Mycoplasma* contamination in this type of study, even when the analyzed immune responder cells are not directly infected. It is noteworthy that even short-term co-culture experiments of less than one day can be perturbed by this crosstalk.

We have investigated the nature of this bystander immune cell activation and demonstrate that the soluble pro-inflammatory factor released from a *Mycoplasma hyorhinis*-infected B16 cell line can be inactivated by protease treatment. In addition, its detection by innate immune cells was blocked by chloroquine treatment of the responder cells. These findings suggest a sensing mechanism that is different from the recognition of canonical *Mycoplasma*-associated lipoproteins by TLR2. Indeed, the activation of TLR2 by both the *Mycoplasma*-derived lipoprotein MALP2 and the synthetic ligand Pam_3_CSK is not affected by chloroquine [27, 28]. We confirm these data with the finding that IL-6 release from GM-CSF DCs in response to *Mycoplasma hyorhinis-*infected B16 cell material was only partially dependent on functional TLR2 signaling. Furthermore, previous studies showed that the N-terminal lipoylated structures of bacterial lipoproteins are vital for their recognition by TLR2 [7]. In line with this, enzymatic degradation of the lipid moiety by lipase treatment but not protease treatment has been shown to impair the pro-inflammatory properties of a *Mycoplasma*-derived lipoprotein [23]. Thus, our findings demonstrate that the culture supernatant from *Mycoplasma hyorhinis*-infected B16 cells contain the TLR2-independent factors we describe alongside with canonical TLR2-dependent ligands; presumably lipoproteins such as MALP-2. Interestingly, previous studies in TLR2-deficient mice have already suggested that the innate immune system is able to recognize *Mycoplasma* through mechanisms that are independent of TLR2, as these animals did not show reduced IL-6 release in comparison to wild-type controls during *Mycoplasma pulmonis* infection [10].

Due to our experimental setup, we cannot exclude transfer of infectious *Mycoplasma hyorhinis* to the immune cell cultures via the B16 supernatant. However, decontamination of the B16 cell culture supernatants by filtration and/or UV irradiation had only a marginal effect on their immunostimulatory capacity. These data demonstrate that this immune response is not based on active infection. Whether the described immunstimulatory factors are of bacterial origin or constitute stress molecules released from B16 cells in response to chronic infection remains to be determined.

Not only cDCs were activated by the factor released from *Mycoplasma hyorhinis*-infected cell lines, but pDCs also similarly responded by producing large amounts of type-I IFNs. Plasmacytoid DCs do not express significant levels of TLR2 but mainly endosomal TLR7 and TLR9, which detect nucleic acids [29]. Both the dependency of cDC activation on MyD88 signaling as well as the inhibition of pDC and cDC activation by chloroquine support the involvement of TLR7 and/or TLR9 in this context. However, the protein factor released from *Mycoplasma hyorhinis*-contaminated cell lines may not necessarily be a ligand for either TLR7 or TLR9, but could rather serve as a stabilizing, chaperone-like factor to nucleic acids. Indeed, cell stress-associated factors such as the high-mobility group box (HMGB) proteins 1 and 2 have been shown to form stable complexes with nucleic acids that facilitate TLR ligation and subsequent production of type-I IFNs and pro-inflammatory cytokines [30, 31]. Furthermore, the antimicrobial peptide LL37 has been shown to bind both host DNA and extracellular RNA released from dying cells [32, 33]. Within these complexes, nucleic acids are protected from degradation and are transported into the endosome, where in pDCs they can trigger TLR-dependent release of type-I IFNs. It will be of interest to investigate whether clinical *Mycoplasma* isolates are associated with similar ligands and whether their recognition plays a role in pathology or protective immunity in *Mycoplasma* infections.

In summary, we demonstrate that *Mycoplasma hyorhinis*-infected cells release factors that are different in sensing, signaling and cytokine output from previously described TLR2 ligands, indicating an involvement of either a novel ligand or non-canonical signaling pathway for the detection of *Mycoplasma hyorhinis* contamination by bystander cells. These data demonstrate a new mechanism by which *Mycoplasma hyorhinis* contamination can affect cell culture experiments. Previous findings showed that chronic *Mycoplasma* infection in a human monocytic cell line can suppress its TLR function [11]. In contrast, we demonstrate here that primary cells, which are shortly co-cultured with contaminated cells, produce pro-inflammatory cytokines and type I interferon, probably through endosomal TLR activation. The precise nature of the ligand remains to be elucidated. In conclusion, *Mycoplasma* contamination perturbs cell culture experiments with immune cells on at least two levels: inhibition of TLR signaling in chronic infection [11] and activation of innate immunity in short-term co-culture experiments. The rigorous control of *Mycoplasma* infection is therefore essential also in primary immune cells to avoid involuntary skewing of experimental results.

## Supporting Information

S1 Fig The immunostimulatory capacity of culture supernatant from *Mycoplasma*-contaminated cells is not specific to the murine B16 melanoma cell line. Freshly isolated murine bone marrow cells were cultured in the presence of either cell lysates or the culture supernatant of one of different *Mycoplasma*-infected murine tumor cell lines (B16 melanoma, CT26 colon carcinoma or EL4 lymphoma). After 18 h, IL-6 levels in the bone marrow cell culture supernatant were measured by ELISA. Data give the mean + S.E.M. of triplicate samples and are representative of at least three independent experiments. Asterisks indicate statistically significant differences to the unstimulated control. SN, supernatant.
(TIF)

S2 Fig Activation of innate immune cells by supernatant from *Mycoplasma*-infected cells is inhibited by chloroquine treatment. **(A)** Bone marrow cells from wild-type mice were cultured in the presence of diluted supernatant from *Mycoplasma hyorhinis*-infected B16 cells. In some conditions, chloroquine was added to the cultures. After 18 h, IL-6 levels in the immune culture supernatants were analyzed by ELISA. **(B)** Murine bone marrow cells or **(C)** GM-CSF DCs were treated with chloroquine or control medium and were cultured in the presence of different defined TLR ligands. After 18 h, IL-6 levels in the immune culture supernatants were analyzed by ELISA. Data give the mean values of triplicate samples + S.E.M. that are representative of two independent experiments. Asterisks indicate statistically significant difference to the appropriate non-inhibitor-treated conditions. ND, not determined.
(TIF)

S3 Fig Irradiation and/or filtration efficiently decontaminate culture supernatants of *Mycoplasma*-infected B16 cells. Non-infected B16 cells were cultured in the presence of supernatant from *Mycoplasma hyorhinis*-contaminated B16 cells and were subsequently passaged twice. Some of the infectious B16 culture supernatant was decontaminated by filtration and/or UV irradiation before transmission to non-infected B16 cells. GM-CSF DCs were then cultured in the presence of culture supernatant from either primarily infected B16 cells (black bars) or B16 cells that had been co-cultured with infectious or decontaminated supernatant (red bars). **(A)** After 18 h, levels of IL-6 in the DC culture were determined by ELISA. Data give the mean values of triplicate samples + S.E.M. **(B)** The scheme gives an overview of the experimental design. SN, supernatant; prim., primary; second., secondary.
(TIF)

S4 Fig Proteinase K treatment of B16 cell lysates results in strongly impaired upregulation of IL-6 mRNA levels in bone marrow cells. Bone marrow cells were cultured in the presence of cell lysates from *Mycoplasma hyorhinis*-infected B16 cells. Some B16 cell lysates were pre-treated with proteinase K. 18 h later, IL-6 mRNA levels in the bone marrow cells were determined by quantitative real-time PCR. Data give the mean values of at least triplicate samples + S.E.M. and are expressed as expression relative to actin mRNA.
(TIF)
