# Supplementary figures and images for: Mycoplasma hyorhinis-Contaminated Cell Lines Activate Primary Innate Immune Cells via a Protease-Sensitive Factor
Source: PLoS One. 2015 Nov 13;10(11):e0142523. doi: 10.1371/journal.pone.0142523 (PMC4643973; doi:10.1371/journal.pone.0142523)

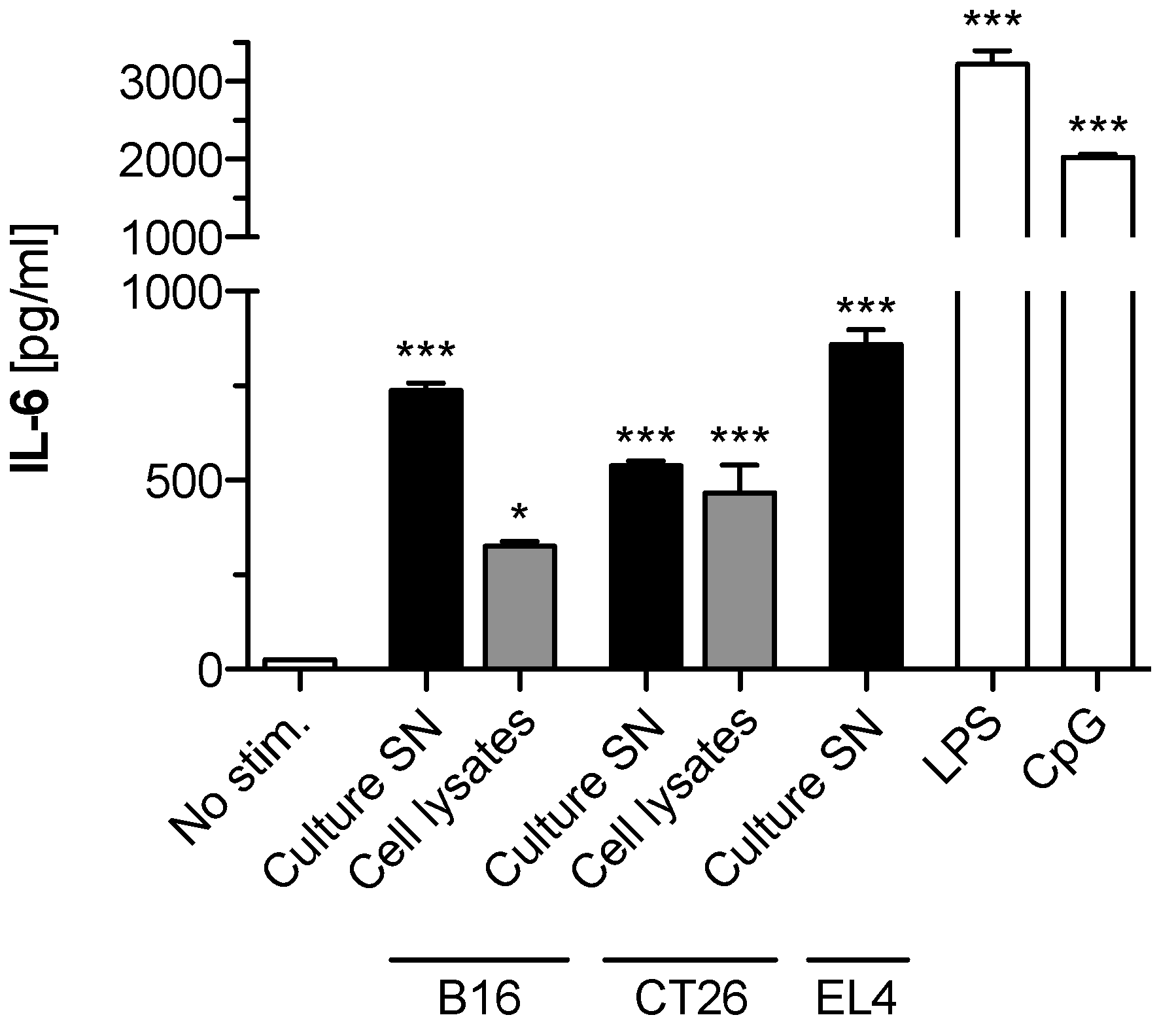

Supplement: S1 Fig — Freshly isolated murine bone marrow cells were cultured in the presence of either cell lysates or the culture supernatant of one of different Mycoplasma-infected murine tumor cell lines (B16 melanoma, CT26 colon carcinoma or EL4 lymphoma). After 18 h, IL-6 levels in the bone marrow cell culture supernatant were measured by ELISA. Data give the mean + S.E.M. of triplicate samples and are representative of at least three independent experiments. Asterisks indicate statistically significant differences to the unstimulated control. SN, supernatant. (TIF) [file pone.0142523.s001.tif]

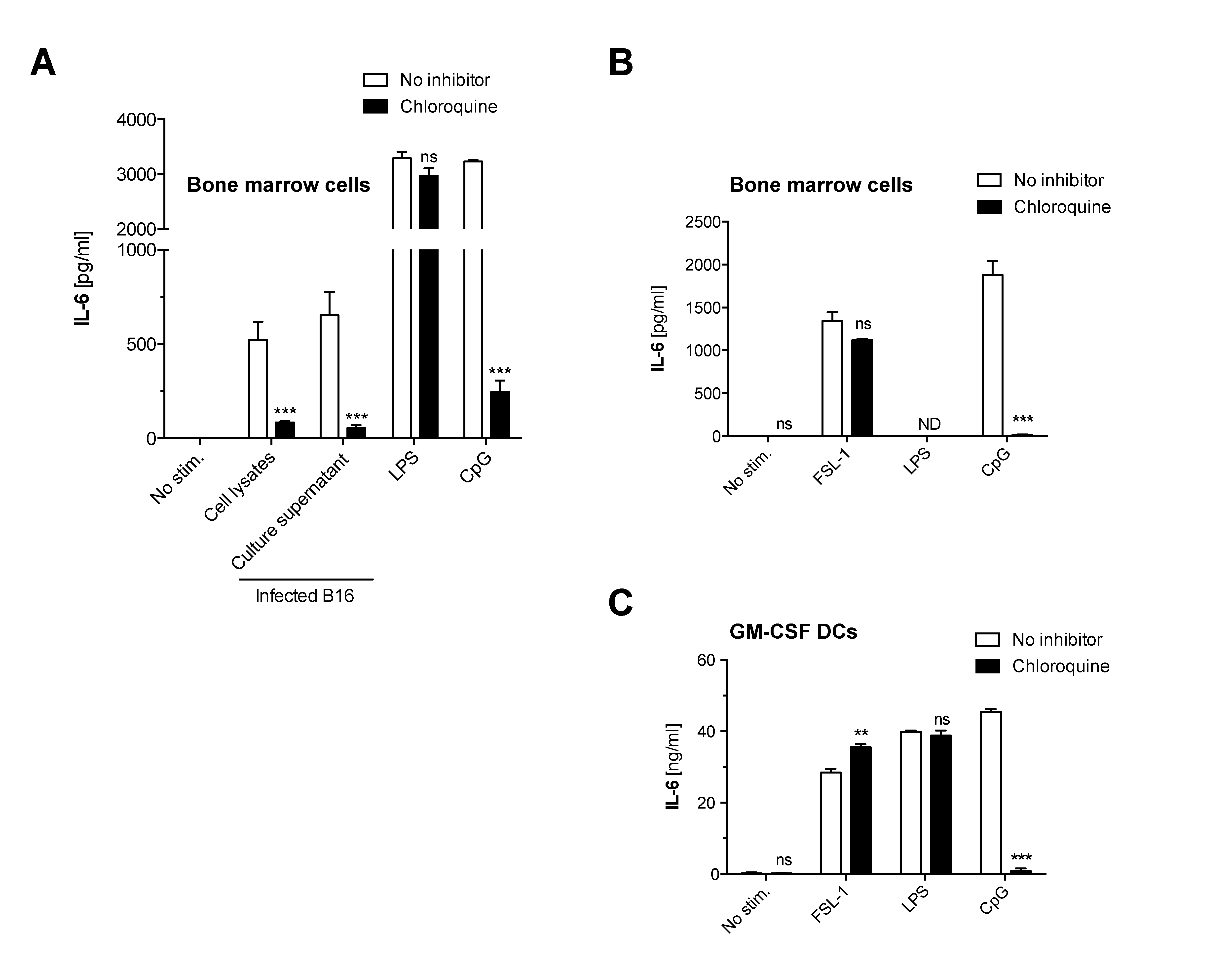

Supplement: S2 Fig — (A) Bone marrow cells from wild-type mice were cultured in the presence of diluted supernatant from Mycoplasma hyorhinis-infected B16 cells. In some conditions, chloroquine was added to the cultures. After 18 h, IL-6 levels in the immune culture supernatants were analyzed by ELISA. (B) Murine bone marrow cells or (C) GM-CSF DCs were treated with chloroquine or control medium and were cultured in the presence of different defined TLR ligands. After 18 h, IL-6 levels in the immune culture supernatants were analyzed by ELISA. Data give the mean values of triplicate samples + S.E.M. that are representative of two independent experiments. Asterisks indicate statistically significant difference to the appropriate non-inhibitor-treated conditions. ND, not determined. (TIF) [file pone.0142523.s002.tif]

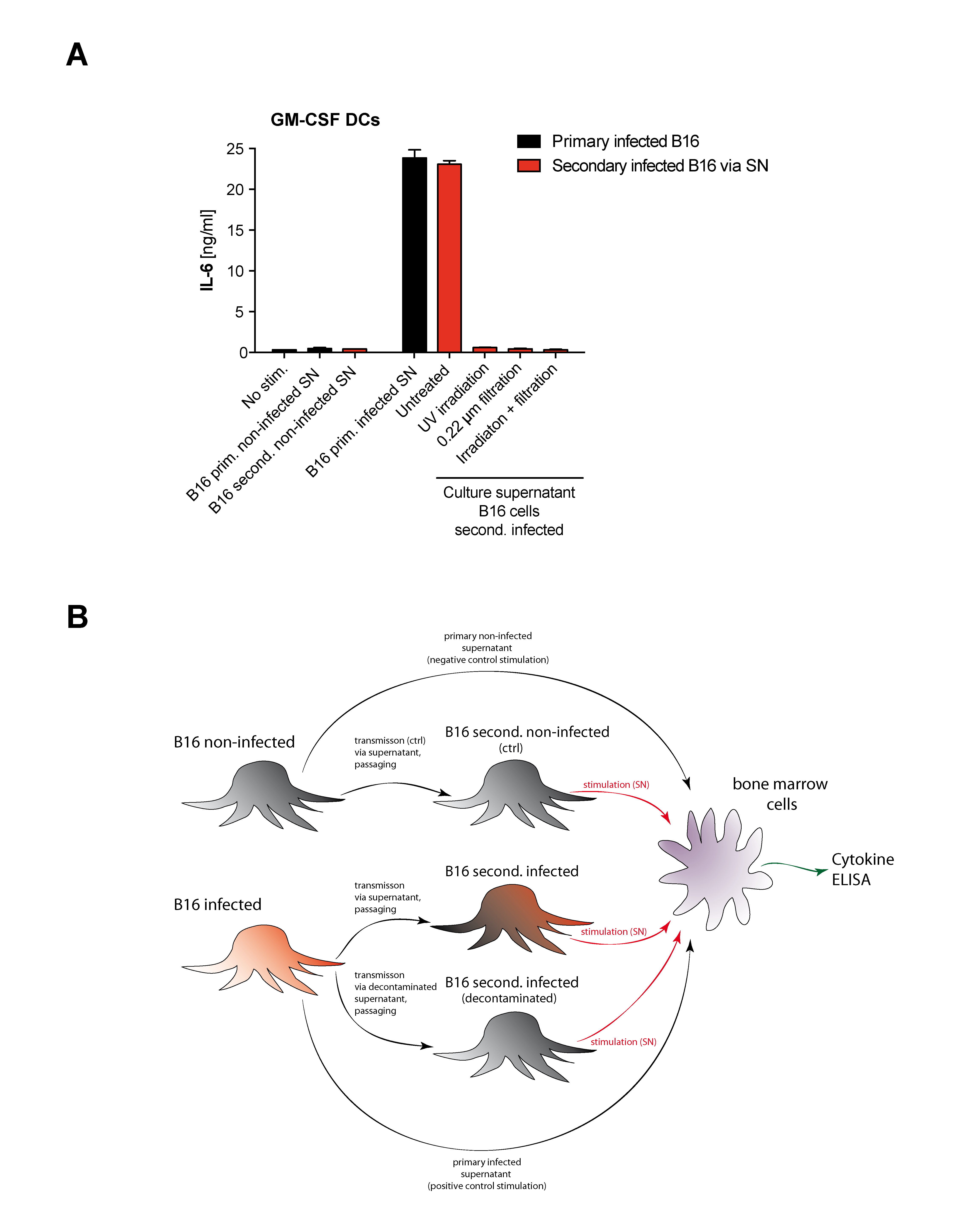

Supplement: S3 Fig — Non-infected B16 cells were cultured in the presence of supernatant from Mycoplasma hyorhinis-contaminated B16 cells and were subsequently passaged twice. Some of the infectious B16 culture supernatant was decontaminated by filtration and/or UV irradiation before transmission to non-infected B16 cells. GM-CSF DCs were then cultured in the presence of culture supernatant from either primarily infected B16 cells (black bars) or B16 cells that had been co-cultured with infectious or decontaminated supernatant (red bars). (A) After 18 h, levels of IL-6 in the DC culture were determined by ELISA. Data give the mean values of triplicate samples + S.E.M. (B) The scheme gives an overview of the experimental design. SN, supernatant; prim., primary; second., secondary. (TIF) [file pone.0142523.s003.tif]

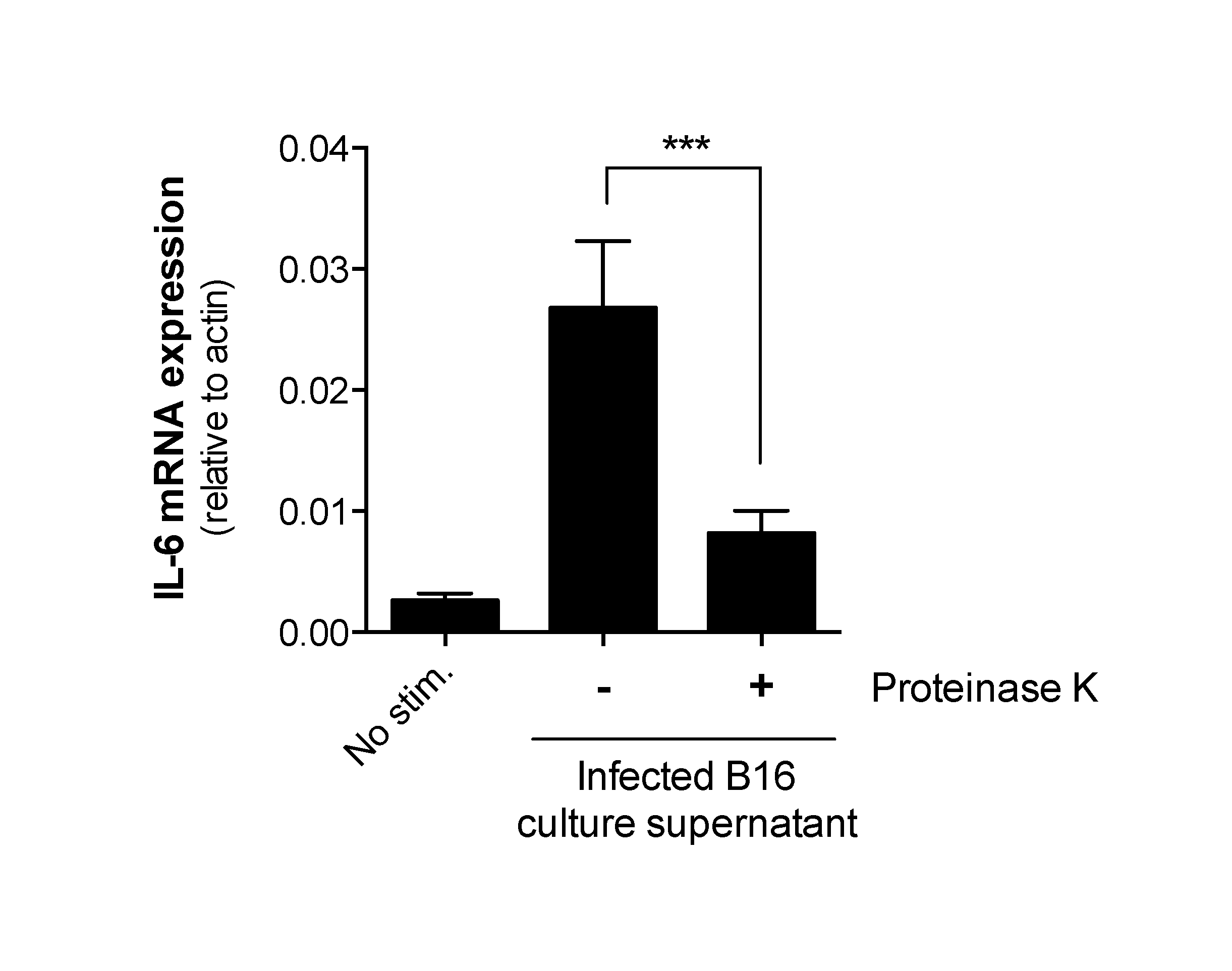

Supplement: S4 Fig — Bone marrow cells were cultured in the presence of cell lysates from Mycoplasma hyorhinis-infected B16 cells. Some B16 cell lysates were pre-treated with proteinase K. 18 h later, IL-6 mRNA levels in the bone marrow cells were determined by quantitative real-time PCR. Data give the mean values of at least triplicate samples + S.E.M. and are expressed as expression relative to actin mRNA. (TIF) [file pone.0142523.s004.tif]
